# *APOE* Genotype-Stratified Meta-Analysis of Cognitive Decline Reveals Novel Loci for Language and Global Cognitive Function in Older Adults

**DOI:** 10.3390/ijms26146940

**Published:** 2025-07-19

**Authors:** Vibha Acharya, Kang-Hsien Fan, Beth E. Snitz, Mary Ganguli, Steven T. DeKosky, Oscar L. Lopez, Eleanor Feingold, M. Ilyas Kamboh

**Affiliations:** 1 Department of Human Genetics, School of Public Health, University of Pittsburgh, Pittsburgh, PA 15261, USA; via16@pitt.edu (V.A.); feingold@pitt.edu (E.F.); 2 Department of Neurology, School of Medicine, University of Pittsburgh, Pittsburgh, PA 15213, USA; snitbe@upmc.edu (B.E.S.); lopezol@upmc.edu (O.L.L.); 3 Department of Psychiatry, School of Medicine, University of Pittsburgh, Pittsburgh, PA 15213, USA; gangulim@upmc.edu; 4 Department of Epidemiology, School of Medicine, University of Pittsburgh, Pittsburgh, PA 15213, USA; 5 McKnight Brain Institute and Department of Neurology, College of Medicine, University of Florida, Gainesville, FL 32610, USA; steven.dekosky@neurology.ufl.edu

**Keywords:** *APOE*-stratified, genome-wide association, neurocognitive domains, attention, visuospatial function, memory, executive function, language, global cognitive function

## Abstract

Apolipoprotein E (*APOE*) allele 4 (*APOE4*), one of the robust genetic risk factors for AD, has also been associated with cognitive decline in terms of memory, executive function, language, and global cognitive function. *APOE* genotype-stratified analysis can help to identify additional genetic loci which might be masked due to a strong effect of *APOE4*. We conducted a genome-wide meta-analysis in *APOE2* carriers, *APOE4* carriers, and *APOE* 3/3 homozygote groups among 2969 non-Hispanic Whites aged ≥ 65 years using slopes of decline over time across five cognitive domains (attention, language, executive function, memory, and visuospatial function) and global cognitive function. We identified novel genome-wide significant associations for decline in global cognitive function in the intergenic region between *RNU7-66P/RNA5SP208* at rs116379916 (*p* = 1.44 × 10^−9^) in the *APOE* 3/3 group and for decline in language in the intergenic region between *LINC0221/DTWD2* at rs13187183 (*p* = 3.79 × 10^−8^) in *APOE4* carriers. A previously reported locus for decline in attention near *RASEF* at rs6559700 (*p* = 9.95 × 10^−9^) was found to be confined to the *APOE* 3/3 group. We also found two sub-threshold significant associations in the *APOE* 2 group for decline in attention (*IL1RL2*/rs77127114; *p* = 8.64 × 10^−8^) and decline in language (*YTHDC2/KCNN2*, rs116191836; *p* = 5.66 × 10^−8^). Our study points to potential biological pathways pertaining to specific domains within each *APOE* genotype group, and the findings suggest that immune-related pathways, plasma levels of polysaturated fatty acids, and bitter taste receptors may play roles in cognitive decline. Our findings enhance the understanding of cognitive aging and provide a framework for future studies.

## 1. Introduction

Older age is characterized by a decline across several cognitive domains, including memory, executive functioning, and language. Age-related cognitive decline begins in early adulthood and becomes more prominent in older age depending on the interplay between genetic and environmental factors [1]. With the population aging worldwide, cognitive impairment poses a growing public health concern. Studies have shown that cognitive aging has a substantial genetic component that increases with age [2,3,4]; thus, understanding the genetic architecture of cognitive aging can help us to identify the underlying biology and devise novel therapeutics.

In the past decade, numerous genetic studies have been conducted on both general cognitive ability and domain-specific abilities to unravel the genetic component of cognitive endophenotypes, leading to the discovery of hundreds of genetic loci [5,6,7]. However, these genetic loci account for only a fraction of heritability across the cognitive phenotypes, and yet a substantial proportion of genetic variance remains unaccounted for [5,8]. It is also possible that genetic loci with large effect sizes, such as *APOE*, obscure genetic loci with weaker effect sizes. Hence, stratified genetic studies might aid in discovering novel genetic signals and explain missing heritability.

Apolipoprotein E (*APOE*) is one of the major genetic risk factors for late-onset sporadic Alzheimer’s disease (AD). While there are several polymorphisms in the *APOE* gene, two SNPs, rs429358 at codon 112 and rs7412 at codon 158, constitute the common three-allele polymorphism (*APOE2* / *E3 /E*4), resulting in six genotypes (*APOE* 2/2, 2/3, 2/4, 3/3, 3/4, and 4/4). This polymorphism has a differential effect on AD risk. Compared to the common *APOE3* allele, *APOE2* is known to impart a protective effect, and *APOE4* increases the risk of AD and other dementia [9,10,11]. *APOE4* has also been associated with age-related cognitive decline in several cognitive domains, such as memory, executive function, language, and global cognitive function across multiple studies [8,12,13]. *APOE4* carriers are also at elevated risk for cognitive decline, possibly due to increased susceptibility to blood–brain barrier breakdown [14,15,16]. Similarly, *APOE2* individuals are found to be relatively protected from cognitive decline [13] and aging [17]. It is possible that several other genetic loci impart the differential effect across different *APOE* genotypes, which could be obscured in non-stratified analysis. Previous studies focusing on *APOE* subgroups have identified novel genetic loci associated with AD, demonstrating that an *APOE*-stratified analysis can help to discover new associations [18,19,20]. To better understand the genetic architecture of cognitive decline, we conducted genome-wide meta-analyses on older adults in three *APOE* genotype groups: *APOE2* carriers (*APOE* 2/2, 2/3), *APOE3* homozygotes (*APOE* 3/3), and *APOE4* carriers (*APOE* 3/4, 4/4). We excluded individuals with the *APOE* 2/4 genotype due to the presence of both risk and protective alleles.

## 2. Results

The study comprised 3021 individuals derived from three longitudinal cohorts, including Gingko Evaluation of Memory (GEM), the Monongahela-Youghiogheny Healthy Aging Team (MYHAT), and the Monongahela Valley Independent Elders Survey (MoVIES). The demographic details across each *APOE* subgroup in each study cohort are shown in Table 1. There was a relatively higher number of women in the MYHAT and MoVIES cohorts across all *APOE* subgroups as compared to the GEM cohort, which had a slightly higher proportion of men in all *APOE* groups. The mean age of the participants was similar in *APOE* subgroups across the studies. GEM individuals were slightly better educated than MYHAT and MoVIES across all the three *APOE* groups. Cognitive phenotypes from 2969 individuals were analyzed, of which 413, 1995, and 561 belonged to the *APOE2* carrier, *APOE* 3/3 homozygous, and *APOE4* carrier groups, respectively. Fifty-two individuals with the *APOE* 2/4 genotype were excluded from analysis in total.

**Figure 1:**
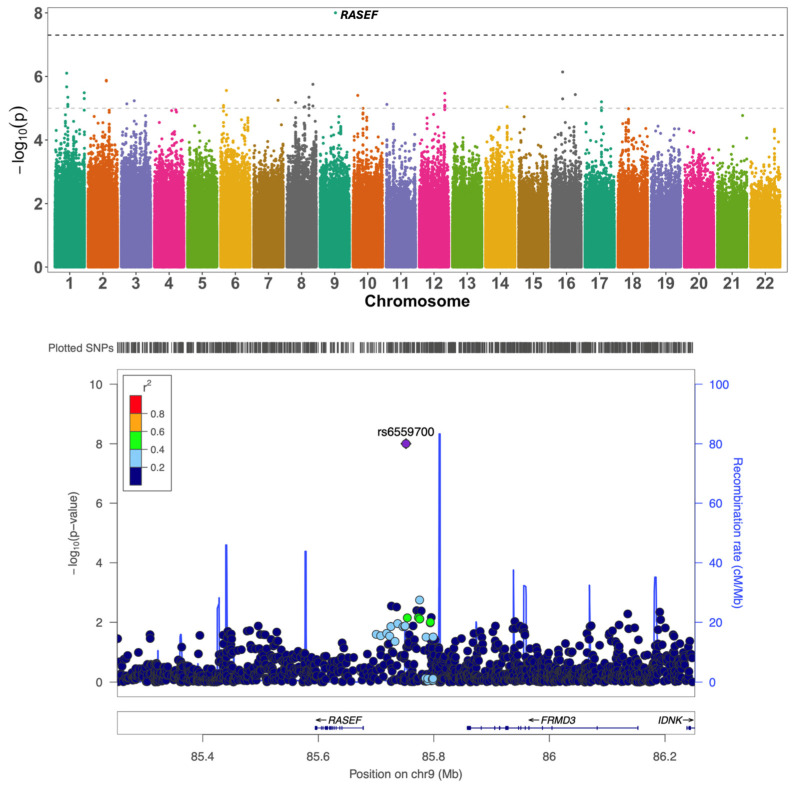
Association of rs6559700 with decline in attention in the *APOE* 3/3 group. (**Top**) Manhattan plot shows the association of rs6559700 with decline in attention in the *APOE* 3/3 group. The black dashed line indicates genome-wide significance (*p* = 5 × 10^−8^), and the gray dashed line indicates suggestive significance (*p* = 1 × 10^−5^). (**Bottom**) Locus Zoom plot of the association of rs6559700 with decline in attention in the *APOE* 3/3 (β = −0.288; *p* = 9.95 × 10^−9^) group.

The QQ plots and the genomic control factor (λ) did not show inflation, indicating that there was no effect of population stratification or cryptic relatedness (Figure S1). We identified three genome-wide significant (GWS) associations. A previously described novel signal for decline in attention [8] on Chr9q21.32 in this sample without *APOE* genotype stratification was found to be confined to the *APOE* 3/3 group at a genome-wide significance (GWS) level (top SNP = rs6559700; MAF = 0.09; β = −0.288; *p* = 9.95 × 10^−9^) (Figure 1). The SNP was also nominally significant for decline in global cognitive function (β = −0.113; *p* = 0.024) in the *APOE* 3/3 genotype group. The Manhattan plots of all the GWAS analyses are presented in the Supplementary Material (Figures S2–S8).

**Figure 2:**
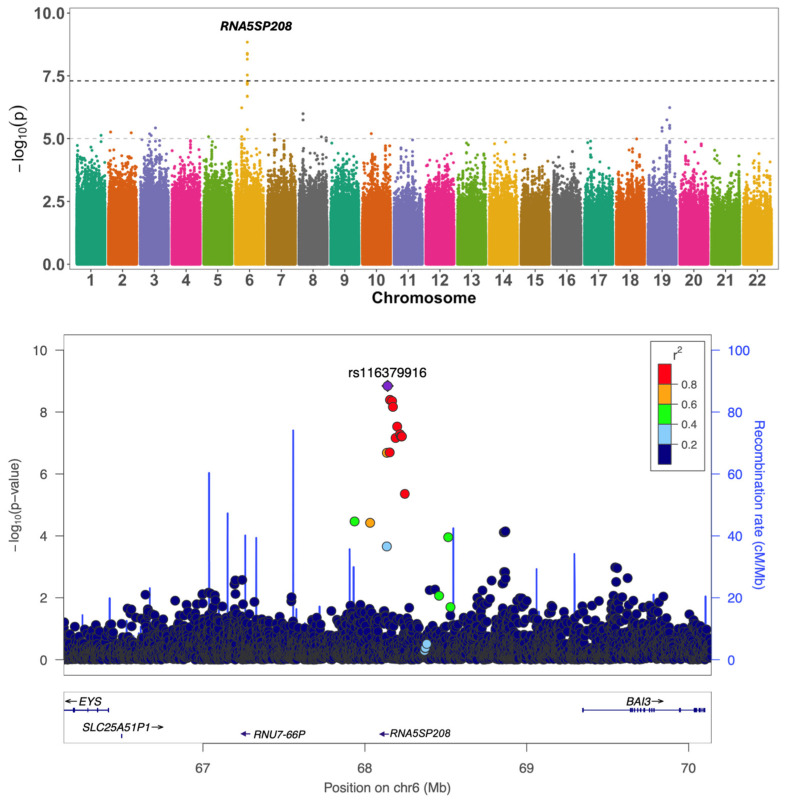
Association of rs116379916 with decline in global cognitive function in the *APOE* 3/3 group. (**Top**): Manhattan plot showing the association of rs116379916 with decline in global cognitive function in the *APOE* 3/3 group. The black dashed line indicates genome-wide significance (*p* = 5 × 10^−8^), and the gray dashed line indicates suggestive significance (*p* = 1 × 10^−5^). (**Bottom**): LocusZoom plot of the association of rs116379916 with decline in global cognitive function in *APOE* 3/3 (β = −0.507; *p* = 1.44 × 10^−9^).

In the *APOE* 3/3 homozygous group, we identified a novel association for decline in global cognitive function on chromosome 6, in the region between *RNU766P* and *RNA5SP208* (top SNP = rs116379916; MAF = 0.03; β = −0.507; *p* = 1.44 × 10^−9^; Figure 2). The SNP was also observed to be nominally associated with all five cognitive domain slopes in the *APOE* 3/3 homozygous group: memory (β = −0.37; *p* = 1.1 × 10^−5^), language (β = −0.33; *p* = 7.19 × 10^−5^), attention (β = −0.30; *p* = 3.73 × 10^−4^), visuospatial function (β = −0.30; *p* = 4.95 × 10^−4^), and executive function (β = −0.23; *p* = 5.90 × 10^−3^). The same signal was also observed at a nominal significance in 2 carriers, but in the opposite direction for the decline in global cognitive function (β = 0.57; *p* = 2.75 × 10^−3^) and decline in visuospatial function (β = 0.64; *p* = 1.77 × 10^−3^). No association of this SNP was found in the *APOE4* group for global cognitive decline (β = 0.1637; *p* = 3.17 × 10^−1^) or any other cognitive domains (Supplementary Table S1).

**Figure 3:**
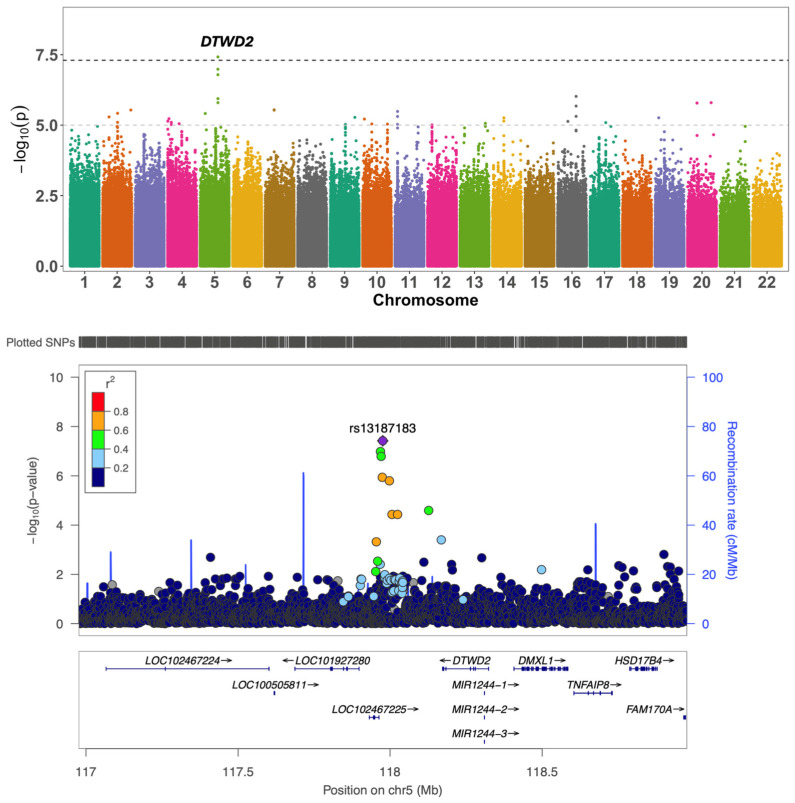
Association of rs13187183 with decline in language in the *APOE4* group. (**Top**) Manhattan Plot showing the association of rs13187183 with the decline in language in the *APOE4* group. The black dashed line indicates genome-wide significance (*p* = 5 × 10^−8^), and the gray dashed line indicates suggestive significance (*p* = 1 × 10^−5^). (**Bottom**) LocusZoom plot of the association of rs13187183 with the decline in language in *APOE4* (β = 0.693; *p* = 3.79 × 10^−8^).

We also observed a novel signal associated with the decline in language in *APOE4* carriers on chromosome 5 in the intergenic region between *LINC02215* and *DTWD2* (top SNP = rs13187183; MAF = 0.056 β = 0.693; *p* = 3.79 × 10^−8^) (Figure 3; Supplementary Table S1). In the *APOE4* group, the top SNP was also nominally associated with global cognitive function (β = 0.56; *p* = 1.46 × 10^−5^), memory (β = 0.42; *p* = 1.35 × 10^−3^), and executive function (β = 0.27; *p* = 2.8 × 10^−2^). Likewise, the top SNP showed a nominal association with the decline in executive function (β = −0.27; *p* = 3.8 × 10^−2^), but in the opposite direction, in the *APOE2* carrier group (Supplementary Table S1). rs13187183 was found to be an eQTL for *HSD17B4* (*p* = 1.92 × 10^−8^), *DTWD2* (*p* = 4.8 × 10^−3^), and *DMXL1* (*p* = 5.2 × 10^−3^) in blood [21].

We also conducted an additional meta-analysis by using the last CDR (0, 0.5, 1) as an additional covariate on the top genome-wide significant findings to test whether the association was driven by MCI/incident dementia. However, we observed a similar allelic effect and significance: rs6559700 was associated with decline in attention in the *APOE* 3/3 group (*p* = 1.38 × 10^−8^, β = −0.28); rs116379916 was associated with decline in global function in the *APOE* 3/3 group (*p* = 2.57 × 10^−9^, β = −0.46), and rs13187183 was associated with the decline in language (*p* = 1.025 × 10^−8^, β = 0.69) in the *APOE4* carrier group, indicating that these are not driven by MCI/incident dementia.

In addition to the three GWS associations, we observed three sub-threshold GWS associations, one in the *APOE4* carrier group and two in the *APOE2* carrier group (Table 2; Figures S9 and S10). In the *APOE4* group, an association with the decline in executive function was observed with an intronic SNP on chromosome 8 in *CSMD1* (top SNP = rs293879; MAF = 0.33; β = 0.32; *p* = 8.49 × 10^−8^). In the *APOE2* group, one association with the decline in attention was with an intronic variant of *IL1RL1* (top SNP = rs77127114; MAF = 0.04; β = 0.86; *p* = 8.64 × 10^−8^) and the other with the decline in language in the intergenic region between *YTHDC2* and *KCNN2* on chromosome 5 (top SNP = rs116191836; MAF = 0.02; β = 1.41; *p* = 5.66 × 10^−8^). Additional variants with *p* < 1 × 10^−6^ are shown in Table 2. All SNPs that surpassed the suggestive threshold of *p* = 1 × 10^−5^ are listed in Supplementary Tables S2–S19, and their FUMA-based annotation is represented in Supplementary Tables S20–S37.

### 2.1. Gene-Based Analysis

In gene-based analyses, we observed some genes with a suggestive significance of *p* < 1 × 10^−5^ (Supplementary Table S38). *ICA1* (*p* = 5.22 × 10^−6^) was found to be associated with the decline in language in the *APOE2* carrier group. Several SNPs (rs6972885, rs35338938, rs11772202, rs6970637) within the gene were also associated with the decline in language in the *APOE2* group. In *APOE2* carriers, *RAB22A,* located on chromosome 20, was found to be associated with the decline in memory (*p* = 5.56 × 10^−6^) and global function (*p* = 8.34 × 10^−5^). *FAM83E* was found to be associated with the decline in global cognitive function (*p* = 7.94 × 10^−6^) in the *APOE* 3/3 homozygous group.

### 2.2. Gene Mapping

We used three techniques to map SNPs to genes: positional, eQTL, and chromatin mapping (Supplementary Tables S39–S56). For the attention domain, there were a total of 65, 118, and 120 genes mapped in the *APOE2* carrier, *APOE* 3/3, and *APOE4* carrier groups, respectively. Some of the genes were mapped using all three techniques (Table 3). For example, for the decline in attention, these included *ZNF608* and *ARID4A* in *APOE2* carriers, *CNN3* and *RP4-639F20.1* in *APOE* 3/3, and *USP36* and *IRF1* in *APOE4* carriers.

### 2.3. Gene Set Enrichment Analysis

Gene set enrichment analysis was conducted using the FUMA-mapped genes for each cognitive domain within each *APOE* group (Supplementary Table S57). In the *APOE4* group, the executive domain was enriched for biological pathways related to the serine phosphorylation of stat protein and immune function, and in the GWAS catalog, it was enriched for genes implicated in autism and alcohol use. There were several distinct as well as shared pathways associated with attention in each *APOE* group. The top pathway associated with attention was the serum protein level (sST2) in the *APOE2* group; in the *APOE3* group, the most significant pathways included prostaglandin secretion and transport. In all three *APOE* groups, the attention domain was enriched for genes associated with inflammatory and autoimmune-related diseases such as asthma, celiac disease, and lupus. Interestingly, in the *APOE4* group, memory was enriched for caffeine consumption, handedness, and low-density lipoprotein and high-density lipoprotein levels and their interaction with diuretics use. In the *APOE2* group, visuospatial and executive functions were enriched for the genes associated with plasma levels of omega-6-polyunsaturated fatty acids (linolenic acid, gamma-linolenic acid, dihomo gamma-linolenic acid). Global function was enriched for bitter taste receptors in the *APOE2* group, and genes associated with carcinoembryonic antigen levels, a serum biomarker of malignancy, were enriched in the *APOE* 3/3 homozygous group. Language function in the *APOE2* group was enriched for genes implicated in chronic venous diseases.

### 2.4. Cognitive Ability and AD

We assessed the association of cognition-associated SNPs that surpassed the suggestive significance threshold (*p* < 1 × 10^−5^) in our study with AD using a recently published large AD meta-GWAS by Bellenguez et al. [22]. Among the 220 SNPs associated with cognitive decline in the *APOE2* group, 217 SNPs were available in recent largest meta-analysis of AD [22] and 24 of them were found to be nominally associated, with *p* values ranging from 0.047 to 0.014 (Supplementary Table S58). In the *APOE* 3/3 group, 174 of 180 SNPs associated with cognitive traits were available in AD study [22] of which 12 were nominally associated with AD, with *p* values ranging from 0.047 to 0.00025 (Supplementary Table S59). Similarly, in the *APOE4* group, 231 of 237 SNPs were available in prior AD study [22], and 16 of those were nominally associated with AD, with *p* values ranging from 0.042 to 0.0055 (Supplementary Table S60).

### 2.5. Comparison with AD-Associated SNPs

We also evaluated the association of top-reported AD SNPs with cognitive domains in each of the three *APOE* groups. Ninety-nine lead SNPs were extracted from Kamboh [9] and Bellenguez et al. [22], of which *p* values were available for 51 SNPs in our cognitive decline data (Supplementary Table S61). AD SNP *GRN*/rs708382 showed the most significant association with decline in executive function (*p* = 8.61 × 10^−4^, β = −0.19) in the *APOE4* group. This SNP was also nominally associated with decline in language (*p* = 9.09 × 10^−3^, β = −0.19), global cognitive function (*p* = 2.5 × 10^−2^, β = −0.15), and visuospatial function (3.9 × 10^−2^, β = −0.15) in the *APOE2* group and with decline in visuospatial function (*p* = 2.3 × 10^−2^, β = −0.07) and attention (*p* = 4.6 × 10^−2^, β = −0.06) in the *APOE* 3/3 homozygous group. As reflected by the β-values, the risk allele of *GRN*/rs708382 was associated with the decline of the above-mentioned domains.

## 3. Discussion

We conducted an *APOE*-stratified genome-wide meta-analysis across three *APOE* groups—*APOE2* carriers, *APOE* 3/3 homozygous, and *APOE4* carriers—in older adults aged 65 and above from three prospective cohorts: MYHAT, MoVIES, and GEMS. Using single-variant, gene-based, and gene set analyses across five neurocognitive domains—attention, memory, executive function, language, visuospatial function—and global cognitive function, we identified genes and biological pathways relevant to domain-specific decline within *APOE* subgroups.

A previously described novel signal for decline in attention on Chr9q21.32 in these cohorts [8] was found to be GWS in the *APOE* 3/3 group only (rs6559700; *p* = 9.95 × 10^−9^). This SNP is an eQTL for *RASEF*, *FRMD3*, and *IDNK* in blood and brain tissues. We identified another novel locus in the *APOE* 3/3 group associated with decline in global cognitive function in the intergenic region between *RNU7-66P* and *RNA5SP208* on chromosome 6. The top SNP, rs116379916, is in linkage disequilibrium (LD) with rs73449416 and rs9363753, which have previously been reported to be associated with cognitive decline in AD [23] and with educational attainment [24], respectively. *RNA5SP208* is a ribosomal pseudogene, and *RNU766P* represents the U7-small nucleolar pseudogene. Recent studies suggest these pseudogenes might be involved in transcriptional regulation and thus might play a role in health and diseases [25,26]. For example, a pseudogene, *ACTBP2*, was found to interact with other genes to increase blood–brain permeability in cellular models of AD, suggesting that pseudogenes could modulate gene expression through epigenetic and transcriptional regulation [27]. Other nearby coding genes include *EYS* and *BAI3*, also known as adhesion G protein-coupled receptor B3 (*ADGRB3*). *EYS* encodes the eye shut homolog gene, a major cause of the recessive condition retinitis pigmentosa. BAI3, a G-protein coupled receptor highly expressed in the brain, modulates synaptic plasticity and is involved in nervous system development, learning, and memory. It has also been implicated in neuropsychiatric illnesses such as schizophrenia and bipolar disorder [28,29,30].

In the *APOE4* group, we observed an association of chr5:117976098 in the intergenic region between *LINCO2215/DTWD2* with the decline in language. The lead SNP, rs13187183, is an eQTL for *HSD17B4*, *DTWD2,* and *DMXL1* in blood. *HSD17B4* encodes a multifunctional enzyme involved in fatty acid beta-oxidation and lipid metabolism in peroxisomes. Mutations in *HSD17B4* are linked to Perrault syndrome, which is associated with hearing loss and intellectual disability, and D-bifunctional protein deficiency, a fatty acid metabolic disorder [31,32]. Compound heterozygous mutations in *HSD17B4* also contribute to middle-age-onset spinocerebellar ataxia, with motor, hearing, and speech impairments [33]. Interestingly, an eQTL variant (rs421765) for *DTWD2* and *DMXL1* has also been associated with severe otitis media (severe ear infection) in Aboriginal Australians [34]. Otitis media frequently leads to conductive hearing loss if not treated promptly. Hearing loss is negatively correlated with language skills in children and older adults [35] and has been associated with cognitive decline across multiple domains, including language and global cognition [36]. As older adults are likely to develop hearing loss with increasing age, it is plausible that genes influencing hearing loss might have a pleotropic effect on decline in language. Recently, age-related hearing loss has been identified as one of the largest modifiable risk factors for dementia, and the use of hearing aids has been found to be associated with reduced cases of dementia [37,38].

We also observed three sub-threshold GWS associations: *CSMD1* with decline in executive function in the *APOE4* group and *IL1RL1* with decline in attention and *YTHDC2-KCNN2* with decline in language in the *APOE2* group. CSMD1, a transmembrane protein involved in various cellular processes, is highly expressed in the brain and linked to diseases like cancer, lupus, and schizophrenia [39]. *CSMD1* has also been reported to be associated with general cognitive ability and executive function in healthy Greek males [40]. *IL1RL1*, also widely known as suppression of tumorigenicity (ST2), codes for the soluble protein sST2 and interleukin-1 receptor-like 1 cytokine receptor, which is a specific receptor for Interleukin-33 (IL-33). IL-33 is a cytokine expressed by astrocytes, oligodendrocytes, and endothelial cells and is involved in neuroimmune remodulation. Mouse models of AD have shown that IL-33/IL1RL1 signaling can alleviate cognitive impairment in AD by activating microglia to phagocytize amyloid beta [41]. This was also further elucidated in a recent AD GWAS, where another *IL1RL1*/rs1921622 variant was found to have protective effects in female *APOE4* carrier participants by suppressing the soluble receptor that inactivates the microglial response to amyloid beta [42]. Soluble receptor beta plasma levels were found to be elevated in female AD patients in that study. sST2 plasma levels were also found to be elevated in middle-aged Chinese participants with metabolic syndrome [43]. In our study, the top *IL1R1*/rs77127114 SNP was mapped to several genes of the IL1 family, such as *IL18R1*, *IL18RAP*, and *IL1R1*, and several variants in this region (rs14465596, rs13405222, rs17639215, rs3752659) were not found to be in LD (r2 = 0.12-0.14) with the reported *IL1RL1/*rs1921622 SNP and were also found to impart protection against decline in attention. *IL1RL1*/rs1921622 was not associated with a decline in attention (*p* = 0.1476) in *APOE2* carriers in our study. It seems likely that *APOE2* carriers have decreased levels of sST2 and thus decreased neuroinflammation, perhaps explaining some of the decreased risk of AD in *APOE2* carriers; however, future studies are needed to test this theory. The third sub-threshold association observed in the intergenic region, *YTHDC2/KCNN2*, has been previously associated with cognition-related traits such as educational attainment [44], decline in visuo-construction [45], and brain shape [46].

Gene-based analysis identified three genes with *p* < 1 × 10^−5^: *ICA1*, *RAB22,* and *FAM83E*. *ICA1* codes for islet cell autoantigen 69 (ICA69), an autoantigen of diabetes mellitus and Sjögren’s syndrome. It was suggestively associated with the decline in language in *APOE2* carriers in both single-variant and gene-based analyses and is involved in modulating APP processing [22]. *RAB22* encodes a GTPase protein belonging to the RAB5 subfamily of GTPases, which are localized to early endosomes, and helps in endosomal trafficking [47]. A guanidine exchange factor of RAB5-GTPases, RIN3, was found to upregulate APP processing and the accumulation of toxic amyloid beta in cellular models of AD [48]. *FAM83E*, associated with global cognitive decline in *APOE* 3/3 individuals, is a tumor suppressor gene linked to posterior cortical atrophy (PCA), which causes visuospatial and language dysfunction due to parietal and occipital cortex degeneration [49,50].

We used gene mapping (positional, eQTL, and chromatin mapping) to prioritize genes associated with cognitive endophenotypes and prioritized multiple genes using all three mapping methods (Table 3). Some of the selected genes were previously reported to be associated with certain phenotypes. For example, *APBB2* was reported to be associated with aging [51], *ZNF608* with preclinical AD [52], *ARID4A* and *ARVCF* with neuropsychiatric diseases [53,54], *CNN3* with epilepsy [55], and *BTBD1O* with ALS [56]. In the *APOE* 3/3 group, genes located on chr2q32.2 (*ASNSD1*, *ORMDL1*, *OSGEPL1*, *OSGEPL1-AS1*, *RP11- 455J20.3*) were found to be mapped by all three methods to the decline in language. In the *APOE2* group, several zinc finger protein-coding genes (*ZNF69*, *ZNF788*, *ZNF20*) were mapped to the decline in visuospatial function. *USP36*, which codes for deubiquitinating enzyme [57], and *IRF1*, which regulates microglia, were prioritized for the decline in attention. *KAT2A*, a transcriptional activator that plays a role in hippocampal memory formation, was mapped to executive function, and regulators of NF-κB and AKT signaling (*PPP2R5A*, *BTBD10*) were mapped to the decline in memory in *APOE4* carriers.

The gene set enrichment analysis indicates that cognitive aging is a complex process with diverse molecular and biological pathways and has both shared and distinct pathways influencing cognitive domains across *APOE* groups. For instance, decline in attention was associated with inflammatory and immune-related pathways across all *APOE* groups, supporting previous studies implicating neuroinflammation in age-related cognitive impairment [58] and neurodegenerative disorders [59]. Neuroinflammation is also considered a risk factor for attention deficit hyperactivity disorder [60], a neurodevelopmental condition characterized by impaired cognitive function in attention and memory. The decline in memory was linked to genes associated with caffeine consumption, but only in *APOE4* carriers; caffeine intake has previously been linked to improved cognitive function [61]. Bitter taste receptor (a G protein coupled receptor) activity was overrepresented in global function in the *APOE2* group. Bitter receptors are also expressed in the brain [62], as well as in the oral cavity (not limited to the tongue), airways, and alimentary canal, and studies have suggested they exert a neuroprotective effect through immunomodulation [63,64]. Executive and visuospatial function in *APOE2* carriers were enriched for genes associated with plasma levels of omega-6 polyunsaturated fatty acids. A recent study found that elevated plasma levels of linolenic acid were suggestively associated with executive function decline in middle-aged healthy participants from Austria [65]. Chronic venous disease-associated genes were linked with decline in language in the *APOE2* group, suggesting the role of vascular health or pathology. Some studies suggest that *APOE2* carriers might be at risk for cerebral angiopathy, despite *APOE2* having a protective effect on aging and AD [66,67].

## 4. Materials and Methods

### 4.1. Study Cohorts

We leveraged 3021 individuals from three different longitudinal studies: GEM [68,69], MYHAT [70,71], and MoVIES [72]. Details about these cohorts have been described previously. In brief, MYHAT and MoVIES were longitudinal, observational, population-based cohorts based in the Monongahela Valley of southwestern Pennsylvania, where participants aged 65 years and older were recruited by age-stratified random sampling from voter registration lists. Beginning in 2006, MYHAT recruited 1982 individuals with sufficient hearing and vision to undertake neuropsychological testing, with decisional capacity to provide informed consent, and not living in nursing homes at study entry. The present study includes MYHAT participants who, at baseline, were free of dementia, with Clinical Dementia Rating (CDR) of 0 (cognitively normal) or 0.5 (mild cognitive impairment), followed annually for six years [6]. Of the 745 MYHAT participants included in the analysis, 24 developed incident dementia and had a CDR score (≥1) at the end visit. MoVIES similarly recruited 1681 participants from 1987 to 1989 with the additional requirement of fluency in English and at least 6th grade education. MoVIES participants included in the current study were free of dementia, with a CDR of 0, throughout the study and were followed every two years for a duration of 12 years. The GEM Study was a randomized control trial comprising 3069 volunteers aged 75 years and above to test the effectiveness of Gingko biloba on the prevention of incident dementia [68]. Participants from four communities (Hagertown, Maryland; Pittsburgh, Pennsylvania; Sacramento, California; and Winston-Salem and Greensboro, North Carolina) in the US participated in the clinical trial for a median period of 6.1 years spanning from 2002 to 2008 [69]. Individuals were assessed every six months to check for incident dementia and were followed annually with a battery of neuropsychiatric tests. The current study includes participants who were free of dementia at the end of the study. Written consent was obtained from participants from all three cohorts and secondary analyses were approved by the Institutional Review Board of University of Pittsburgh. In all three cohorts, participants were tested on a range of tests across five cognitive domains: attention, memory, language, visuospatial function, and executive function. Only participants with non-Hispanic White ancestry, with at least two cognitive assessments, and who consented to genotyping were included in the analysis. Written informed consent was obtained from participants in all three cohorts and secondary analyses were approved by the Institutional Review Board of University of Pittsburgh.

### 4.2. Cognitive Assessment

Neurocognitive tests were focused on five cognitive domains, attention, visuospatial function, executive function, language, and memory, as described previously [6,8]. For each neurocognitive test, the test scores were transformed to z-scores using the baseline mean and standard deviation. For each participant, domain z-scores were derived by averaging the test scores within the specific domain. We also devised a global cognitive function score as the average of all test z-scores in participants who did not miss more than one test score.

### 4.3. Cognitive Decline Slope

A cognitive trajectory was estimated for each person for each domain and global cognitive function by fitting a linear mixed-effect model with random slope and intercept. Individual-specific slopes were extracted, adjusting for the fixed effect of education, sex, and baseline age, as described previously [8]. Since some individuals showed rapid decline, slopes were ranked [0.1,0.99] and transformed using inverse standard transformation in R version 4.3.1, (accessed on 16 December 2023) (https://www.r-project.org), as described previously [6]. Rank normal transformation helps to conform the data to normality. The cognitive decline slopes for the MoVIES cohort were obtained from the previous analysis [6]. These cognitive decline slopes in each domain and global cognitive functions were used to conduct genome-wide associations in each study cohort.

### 4.4. Genotyping, Imputation, and Quality Control

DNA was extracted from buffy coats derived from blood, and genome-wide genotyping was conducted using the Illumina Infinium Multi-Ethnic Global, (Illumina, Incorporation, San Diego, CA, USA), the Illumina Omni2.5 (Illumina, Incorporation, San Diego, CA, United States), and the Omni1-Quad Chips (Illumina, Incorporation, San Diego, CA, USA), in the GEM, MYHAT, and MoVIES cohorts, respectively. In the sample-level quality control (QC), individuals were assessed for relatedness within and across cohorts using identity by descent in PLINK [73]; related individuals were excluded from the analyses. Individuals with a genotyping rate < 95% and mismatched sex and race were also excluded from the analyses. For the SNP-level quality measures, SNPs with Hardy–Weinberg equilibrium (*p* < 1 × 10^−6^), minor allele frequency (MAF < 0.01), and genotyping rate < 95% were not included in the analysis. Imputation was conducted on the Michigan Imputation server using the Haplotype Reference panel version 1.1, and SNPs with r2 < 0.3 were excluded from the analysis. *APOE* genotyping was based on the TaqMan assay (Themo Fisher Scientific; Waltham, MA, USA) and has been described previously [6,74].

### 4.5. Genome-Wide Association and Meta-Analysis

A genome-wide association study (GWAS) was performed on intra-individual domain-specific slopes from five cognitive domains and for global cognitive function using an additive genetic model in PLINK for each study cohort in the three *APOE* groups. Genetic principal components were calculated using SNPs with an MAF > 5%, and the first four genetic principal components (PCs) were added as covariates in the linear regression model. After conducting a GWAS on each cognitive phenotype in each study cohort, the mean effect across the three cohorts was evaluated by performing a fixed-effect inverse variance-based meta-analysis in METAL (Meta-Analysis of Genome-wide Association; http://www.sph.umich.edu/csg/abecasis/metal/, (accessed on 15 January 2024) [75]. The SNPs that were available across all cohorts were used for meta-analysis. The genome-wide threshold was defined as *p* < 5 × 10^−8^, and the suggestive threshold was defined as *p* ≤ 1 × 10^−5^.

### 4.6. Genomic Risk Locus Characterization

Genomic risk loci were defined based on ANNOVAR (https://annovar.openbioinformatics.org/, (accessed on 17 January, 2024) [76] annotation using the SNP2GENE function in Functional Mapping and Annotation of Genetic Associations (FUMA) [77]. Lead SNPs were defined within a genomic risk locus if these surpassed a suggestive threshold of 1 × 10^−5^ and were in linkage disequilibrium (LD) r2 < 0.1 with other SNPs within a block of 250 kilobases (kb). CADD scores, Regulome DB (RDB) scores, and 15 chromatin states were obtained from FUMA. Based on numerous annotations, CADD-ranked scores represent the deleteriousness of an SNP, with a higher score representing higher deleteriousness. A CADD score of 10 represents the top 10% of the deleterious variants, and a CADD score of 20 represents the top 1% among all reference SNVs.

### 4.7. Gene Mapping

We also mapped the SNPs to genes based on positional mapping, eQTL mapping, and chromatin mapping. An SNP was mapped to a gene if it resided 10 kilobases upstream or downstream of a gene. Gene-Tissue Expression version 8 (GTEx), as well as other blood and brain tissues available in FUMA, were used at FDR (*p*) < 1 × 10^−3^ to identify genes whose expression was associated with the SNPs. Additionally, an SNP was mapped to a region if the locus interacted with another region within 250 base pairs (bp) upstream and 500 bp downstream of the transcription start site in HiC adult cortex [78] at FDR < 1 × 10^−6^. The GENE2FUNC function in FUMA was used to find the functional relevance of these mapped genes.

### 4.8. Gene-Based Analysis

MAGMA gene-based and gene set analysis was conducted in FUMA using the SNP2GENE function. SNPs were assigned to 18106 protein-coding genes based on Ensemble build 92 within the 8 kb window upstream and downstream, and genome-wide significance was defined as 0.05/18106 = 2.762 × 10^−6^. For the MAGMA gene set analysis, gene sets from the Molecular Signature (MsigDB) database were extracted from FUMA, and Bonferroni correction was applied to adjust for multiple testing.

## 5. Conclusions

*APOE*-stratified analyses have enabled us to identify novel loci for the decline in language in the *APOE4* group and decline in global cognition in the *APOE* 3/3 group. Our study points to potential biological pathways pertaining to specific domains within each *APOE* genotype group, and the findings suggest that immune-related pathways, plasma levels of polysaturated fatty acids, and bitter taste receptors may play some roles in cognitive decline. These findings deepen our understanding of the biological mechanism of cognitive aging and offer a foundation for future research. Studies examining cognitive traits within *APOE*-stratified groups may yield deeper insights into how gene–environment interactions influence cognitive function. For example, the impact of lifestyle factors such as alcohol and coffee consumption, as well as metabolites like omega-6 fatty acids, can be evaluated across different *APOE* genotypes to assess their roles in cognitive decline.

Our study has several strengths. Given the longitudinal nature of our study cohorts, with participants followed over several years, our study is well-positioned to capture the biological processes underlying cognitive aging, offering advantages over cross-sectional designs. To our knowledge, this is the most comprehensive study conducted to date across neurocognitive domains in *APOE* subgroups, providing domain-specific findings in each *APOE* group. However, the study also has some limitations. Since our study is based on non-Hispanic Whites, further studies need to be conducted in other ancestral cohorts to determine whether the allelic effects are similar across different populations. Additionally, we used linear modeling to phenotype cognitive aging and assumed that cognitive aging follows a linear trend; however, cognitive decline could also follow non-linear trends. Although a small sample size owing to stratification into *APOE* subgroups is another limitation, the stratified analyses have still enabled us to detect association signals obscured in the combined analysis. Thus, our study has elucidated that *APOE*-stratified analyses can aid in gene discovery, and future cognitive genetic studies can be conducted in other populations following this design to corroborate our findings.

## Figures and Tables

**Table 1:** Demographic profile of GEM, MYHAT, and MoVIES stratified by *APOE*.

	Gingko Evaluation of Memory (GEM)	Monongahela-Youghiogheny Healthy Aging Team (MYHAT)	Monongahela Valley Independent Elders Survey (MoVIES)
Total Participants	1898	745	378
Age (Mean (SD))	78.13 ± 3.04	77.15 ± 7.32	77.46 ± 4.23
Female (N, %)	840 (44.25)	451 (60.53)	254 (67.19)
Education (Years)	14.4 (2.9)	13.04 (2.51)	11.81 (2.27)
Baseline CDR, (N, %)			
0	1263 (66.54)	570 (76.51)	378 (100)
0.5	635 (33.45)	175 (23.48)	0 (0)
Last CDR, (N, %)			
0	939 (49.47)	501 (67.24)	378 (100)
0.5	959 (50.52)	220 (29.53)	0 (0)
≥1	0 (0)	24 (3.22)	0 (0)
*APOE2* (N)	258	100	55
Age (Mean (SD))	78.45 ± 3.25	79.02 ± 7.59	77.70 ± 5.16
Female (N, %)	118 (45.73)	52 (52)	46 (95)
Education (Years)	14.19	13	11.89
Baseline CDR (N, %)			
0	162 (62.79)	80 (80)	55 (100)
0.5	96 (37.2)	20 (20)	0 (0)
≥1			0 (0)
Last CDR (N, %)			
0	128 (49.61)	70 (70)	55 (100)
0.5	130 (50.38)	28 (28)	0 (0)
≥1	0 (0)	2 (2)	0 (0)
*APOE* 3/3 (N)	1237	498	260
Age (Mean (SD))	78.18 ± 3.06	77.22 ± 7.31	77.49 ± 4.18
Female (%)	531 (42.92)	305 (61.24)	169 (65)
Education (years)	14.40	13.02	11.73
Baseline CDR (N, %)			
0	840 (67.90)	382 (76.7)	260 (100)
0.5	397 (32.09)	116 (23.2)	0 (0)
≥1	0 (0)	0 (0)	0 (0)
Last CDR (N, %)			
0	615 (49.71)	340 (68.3)	260 (100)
0.5	622 (50.27)	145 (29.1)	0 (0)
≥1	0 (0)	13 (2.6)	0 (0)
*APOE4* (N)	367	138	56
Age (Mean (SD))	77.74 ± 2.65	75.71 ± 7.02	76.96 ± 3.37
Female (N, %)	174 (47.70)	88 (63.76)	34 (60.71)
Education (years)	14.48	13.21	12.17
Baseline CDR (N, %)			
0	238 (64.9)	102 (73.9)	56 (100)
0.5	129 (35.1)	36 (26.1)	0 (0)
≥1	0 (0)	0 (0)	0 (0)
Last CDR (N, %)			
0	181 (49.3)	84 (60.9)	56 (100)
0.5	186 (50.7)	46 (33.3)	0 (0)
≥1	0 (0)	8 (5.8)	0 (0)

Note: CDR = Clinical Dementia Rating.

**Table 2:** List of top and suggestive SNPs with *p* < 1 × 10^−6^ associated with cognitive domains and global cognitive function in three *APOE* groups (*APOE2*, *APOE* 3/3 and *APOE4*). Bold font indicates the genome-wide significant loci.

*APOE* Subgroups	Domain	SNP	Chr:Position	A1/A2	Meta-Analysis			Loc	Gene
					MAF	Beta	*p*		
*APOE4* Group	Attention	rs62371993	5:42622849	C/A	0.04	−0.77	1.87 × 10^−7^	intronic	*GHR*
		rs7320036	13:85848704	C/T	0.14	0.42	4.44 × 10^−7^	intergenic	*LINC00375*, *LINC00351*
		rs62253001	3:69767057	G/A	0.07	0.57	6.60 × 10^−7^	intergenic	*FRMD4B*, *MITF*
		rs4732363	7:138990811	T/C	0.49	0.28	8.27 × 10^−7^	UTR3	*UBN2*
	Executive Function	rs293879	8:4586377	T/G	0.32	0.32	8.49 × 10^−8^	intronic	*CSMD1*
		rs62499971	8:20125115	T/C	0.19	−0.36	2.01 × 10^−7^	intronic	*LZTS1*
		rs2943674	1:85105006	C/T	0.10	−0.45	7.31 × 10^−7^	intergenic	*LINC01555*, *SSX2IP*
	Memory	rs72845557	17:60458122	C/A	0.12	0.46	6.60 × 10^−7^	intronic	*EFCAB3*
	Language	rs13187183	5:117976098	C/T	0.06	0.69	3.79 × 10^−8^	intergenic	*LINC02215*, *DTWD2*
		rs4888647	16:73748328	C/T	0.09	−0.48	9.52 × 10^−7^	intergenic	*LINC01568*, *LOC101928035*
	Global	rs62297316	4:5376604	G/C	0.02	1.21	1.58 × 10^−7^	intronic	*STK32B*
		rs12746598	1:240305899	T/C	0.34	−0.30	9.81 × 10^−7^	intronic	*FMN2*
*APOE* 3/3 Group	Attention	rs6559700	9:85751913	A/G	0.09	−0.29	9.95 × 10^−9^	intergenic	*RASEF*, *FRMD3*
		rs77848581	16:26919840	C/T	0.01	0.71	7.23 × 10^−7^	intergenic	*HS3ST4*, *C16orf82*
	Executive Function	rs62320280	4:131795996	T/C	0.13	−0.23	5.19 × 10^−7^	intergenic	*LINC02479*, *SNHG27*
		rs112356643	13:55089341	T/C	0.02	−0.50	6.99 × 10^−7^	intergenic	*MIR1297*, *MIR5007*
	Memory	rs3865460	19:41447928	G/A	0.21	0.18	5.35 × 10^−7^	lncRNA intronic	*CYP2B7P*
		rs151090836	7:38728382	C/T	0.03	−0.46	5.56 × 10^−7^	intergenic	*FAM183BP*, *VPS41*
		rs426171	13:110557648	C/A	0.35	−0.15	6.04 × 10^−7^	intergenic	*IRS2*, *LINC00396*
		rs116725560	4:8374858	T/C	0.05	−0.35	7.73 × 10^−7^	intronic	*ACOX3*
	Visuospatial Function	rs58302405	2:218000504	T/C	0.03	−0.46	2.69 × 10^−7^	intergenic	*LINC01921*, *DIRC3-AS1*
		rs62521843	8:124198306	A/G	0.03	−0.44	5.17 × 10^−7^	intronic	*FAM83A*
		rs139851690	10:2600111	C/T	0.02	0.56	9.48 × 10^−7^	intergenic	*LINC02645*, *LOC101927824*
	Global Function	rs116379916	6:68140492	T/G	0.03	−0.51	1.44 × 10^−9^	intergenic	*RNU7-66P*, *RNA5SP208*
*APOE2* Group	Attention	rs77127114	2:103000000	A/T	0.04	0.86	8.64 × 10^−8^	intronic	*IL1RL1*
		rs72706424	14:93522585	A/G	0.05	0.71	2.17 × 10^−7^	intronic	*ITPK1*
		rs11465596	2:103000000	A/C	0.10	0.53	4.67 × 10^−7^	intronic	*IL18R1*
		rs11787153	8:139697286	C/T	0.25	0.36	8.22 × 10^−7^	intronic	*COL22A1*
		rs4541656	5:123971932	A/C	0.34	0.33	9.84 × 10^−7^	downstream	*ZNF608*
	Executive Function	rs35871159	7:30781232	T/C	0.36	0.33	2.97 × 10^−7^	intergenic	*CRHR2*, *INMT*
		rs13339093	16:78619484	C/T	0.06	0.69	3.65 × 10^−7^	intronic	*WWOX*
		rs16921936	12:19924660	G/T	0.04	−0.81	3.94 × 10^−7^	intergenic	*AEBP2*, *LINC02398*
	Language	rs116191836	5:113000000	T/C	0.02	1.41	5.66 × 10^−8^	intergenic	*YTHDC2*, *KCNN2*
	Visuospatial Function	rs10849223	12:5384521	C/T	0.14	0.51	2.98 × 10^−7^	intergenic	*LINC02443*, *NTF3*
		rs12319445	12:92357858	G/A	0.02	−1.14	3.30 × 10^−7^	intergenic	*DCN*, *LINC01619*
	Global Function	rs75168743	14:44488668	C/T	0.07	0.63	1.65 × 10^−7^	intergenic	*NONE*, *FSCB*
		rs7998149	13:79077882	G/A	0.48	−0.34	1.85 × 10^−7^	ncRNA intronic	*OBI1-AS1*
		rs12641677	4:182290596	G/A	0.10	−0.52	4.25 × 10^−7^	intergenic	*LINC02500*, *TEMN3-AS1*

**Table 3:** Number of genes mapped for each cognitive trait across domains using positional, eQTL, and chromatin mapping (given in numbers) and genes mapped using all three mapping methods (given by names).

Domain	*APOE2* Carriers	*APOE* 3/3	*APOE4* Carriers
Attention	65 *ZNF608*, *ARID4A*	118 *CNN3*, *RP4-639F20.1*	120 *USP36*, *IRF1*
Executive Function	82 - *	81 *CTB-129O4.1*, *PDP1*	150 *LZTS1*, *KAT2A*, *RAB5C*, *ARVCF*, *DGCR8*
Memory	74 *VANGL1*, *PTPN14*	38 *APBB2*	153 *PPP2R5A*, *TMEM206*, *BTBD10*
Language	78 *MSRA*, *CTB113P19.3*	64 *ASNSD1*, *OSGEPL1*, *OSGEPL1-AS1*, *ORMDL1*, *PRR5L*	89 *PDE6B*
Visuospatial Function	147 *ZNF69*, *ZNF788*, *ZNF20*, *RSL24D1P8*	41 -	122 *PIGCP1*, *CSTF3*, *HIPK3*, *SAMD4A*
Global Function	88 -	95 -	79 -

* Note: Dash (-) indicates no genes were mapped using all three mapping methods.

## Data Availability

The summary statistics generated during the study is available upon request to the senior author.

## Supplementary Materials

The following supporting information can be downloaded at https://www.mdpi.com/article/10.3390/ijms26146940/s1.

## Abbreviations

The following abbreviations are used in this manuscript

AD	Alzheimer’s Disease
APOE	Apolipoprotein E
CDR	Clinical Dementia Rating
MYHAT	The Monongahela-Youghiogheny Healthy Aging Team
MoVIES	Monongahela Valley Independent Elders Survey
GEM	Gingko Evaluation of Memory
LD	Linear Disequilibrium
MAF	Minor Allele Frequency
