# Supplementary material for: APOE Genotype-Stratified Meta-Analysis of Cognitive Decline Reveals Novel Loci for Language and Global Cognitive Function in Older Adults
Source: Int J Mol Sci. 2025 Jul 19;26(14):6940. doi: 10.3390/ijms26146940 (PMC12295717; doi:10.3390/ijms26146940)
Supplement: Supplementary file 1 [file ijms-26-06940-s001.zip › Supplementary Figures Revised.pdf]

Supplementary Figures

APOE Genotype-Stratified Meta-Analysis of Cognitive Decline Reveals Novel Loci for Language and Global Cognitive Function in Older Adults

Vibha Acharya [1], Kang-Hsien Fan [1], Beth E. Snitz [2], Mary Ganguli [3,4], Steven T. DeKosky [5], Oscar L. Lopez [2], Eleanor Feingold [1] and M. Ilyas Kamboh [1,3,*]

[1] Department of Human Genetics, School of Public Health, University of Pittsburgh Pittsburgh, PA 15261, USA; via16@pitt.edu (V.A.); frank.fan@pitt.edu (K.-H.F.); feingold@pitt.edu (E.F.)

[2] Department of Neurology, School of Medicine, University of Pittsburgh, Pittsburgh, PA 15213, USA; snitbe@upmc.edu (B.E.S.); lopezol@upmc.edu (O.L.L.)

[3] Department of Psychiatry, School of Medicine, University of Pittsburgh, Pittsburgh, PA 15213, USA; gangulim@upmc.edu

[4] Department of Epidemiology, School of Medicine, University of Pittsburgh, Pittsburgh, PA 15213, USA

[5] McKnight Brain Institute and Department of Neurology, College of Medicine, University of Florida, Gainesville, FL 32610, USA; steven.dekosky@neurology.ufl.edu

* Correspondence: kamboh@pitt.edu; Tel.: +1-412-624-3021

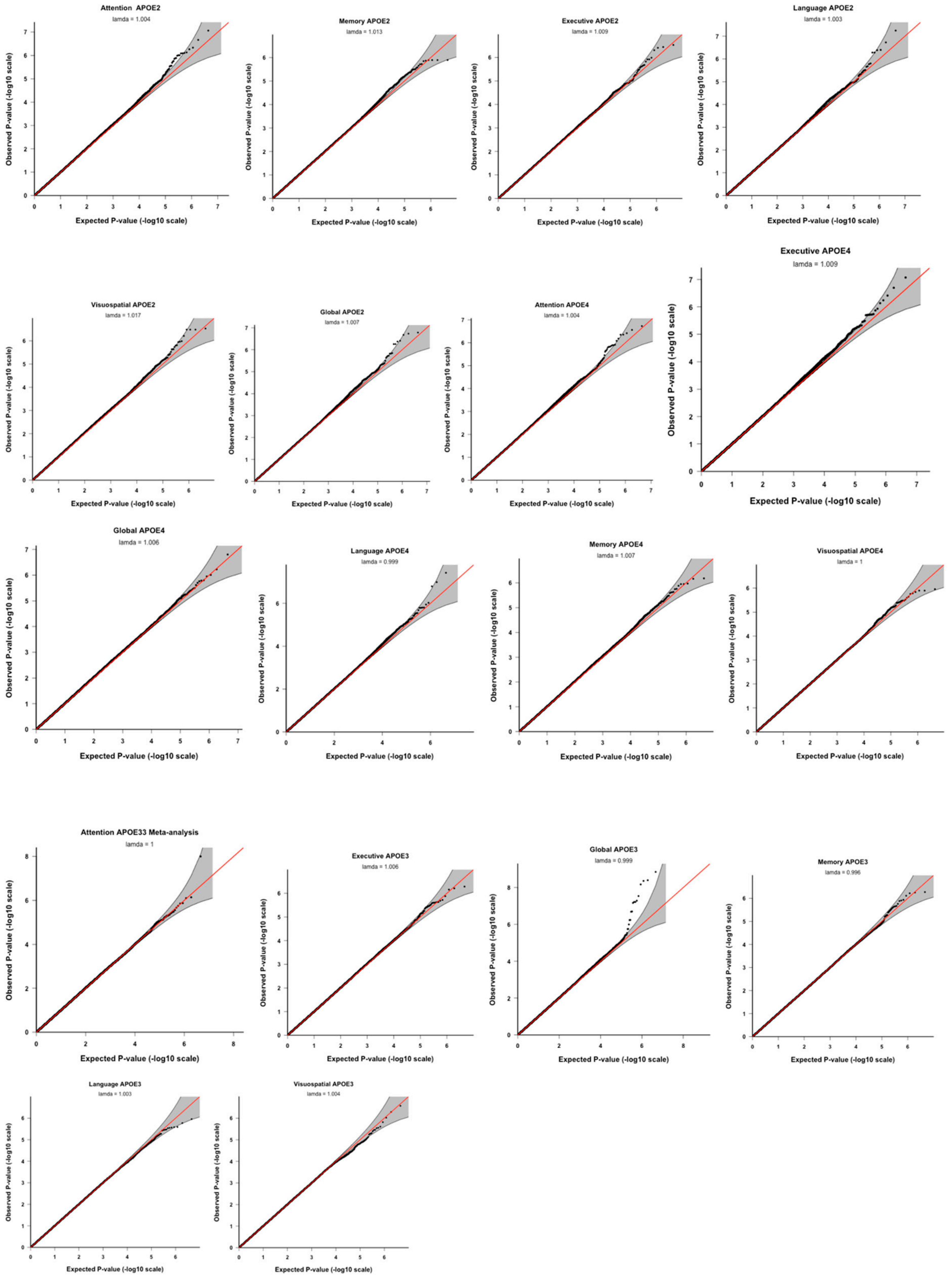

Figure S1 : QQ plots of genome-wide meta-analysis conducted on five cognitive domains and global cognitive function in 3 *APOE* groups(*APOE*2, *APOE* 3/3 and *APOE*4).

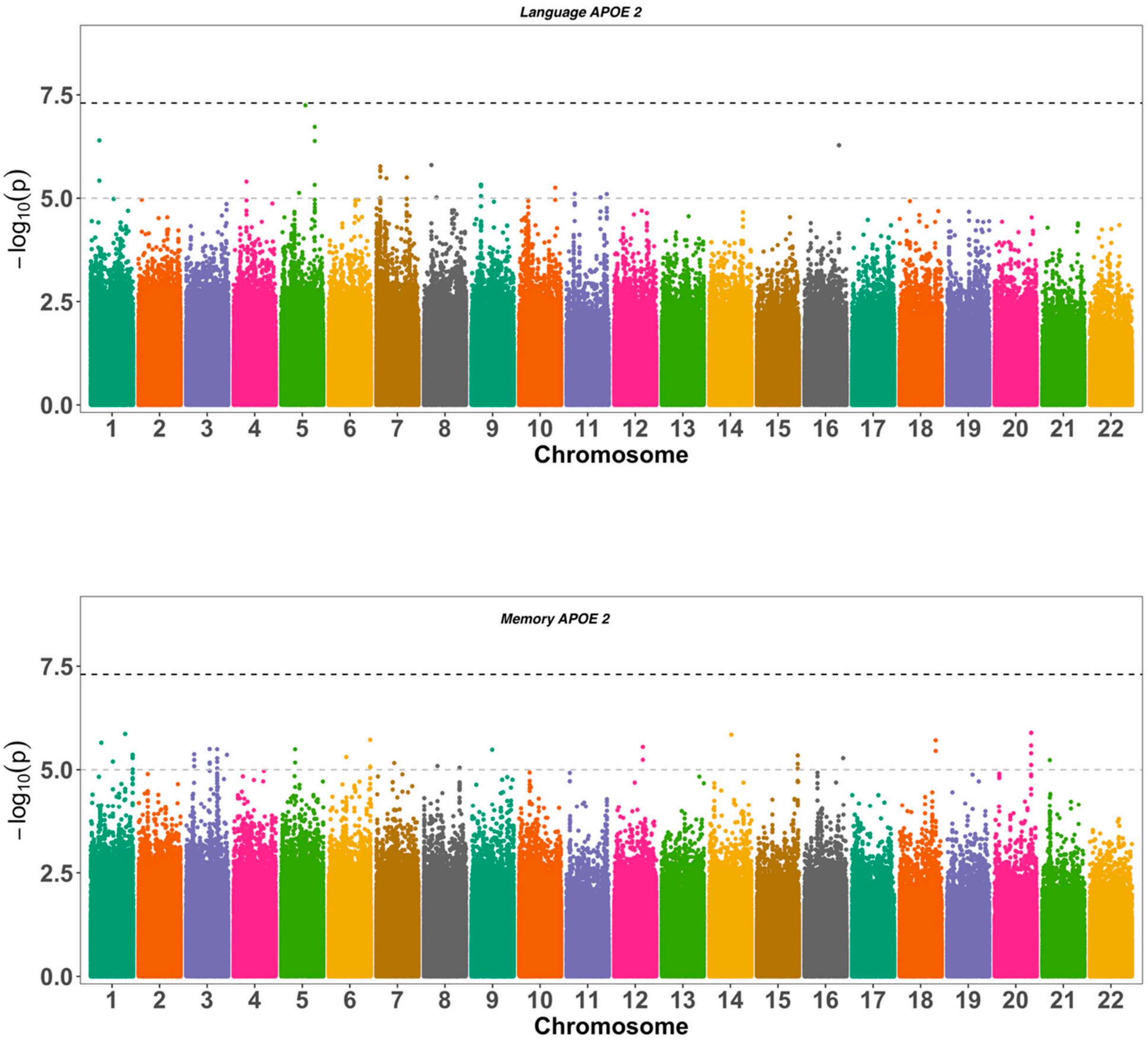

Figure S2: Manhattan plot showing the association of SNPs with the decline of language (top) and memory(below) in *APOE*2 group. The black dash line indicates the genome-wide significance (p=5.E-08) and grey dash line indicates the suggestive significance of (p=1E-05).

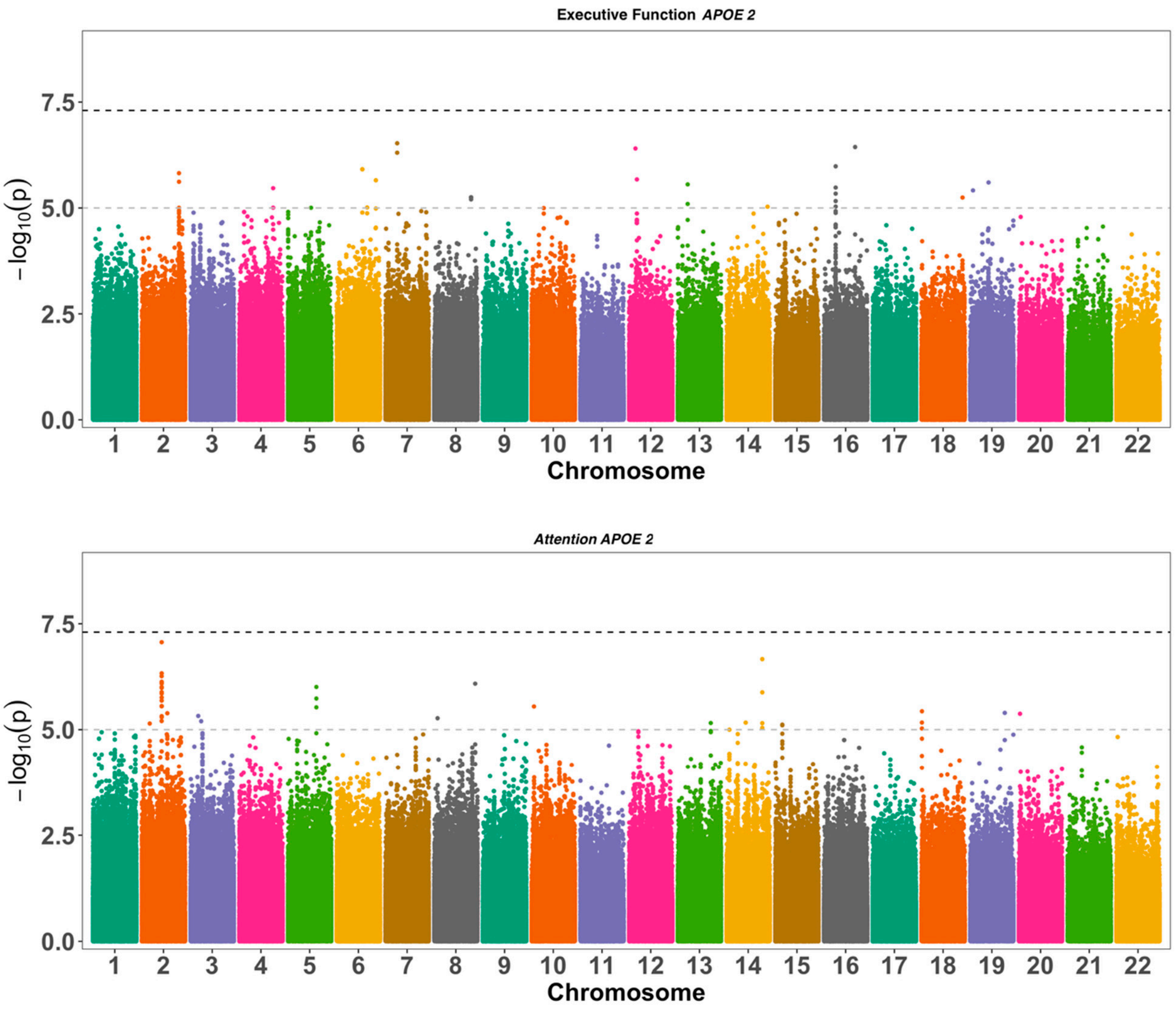

Figure S3: Manhattan plot showing the association of SNPs with the decline of executive function (top) and attention (below) in *APOE*2 group. The black dash line indicates the genome-wide significance (p=5.E-08) and grey dash line indicates the suggestive significance of (p=1E-05).

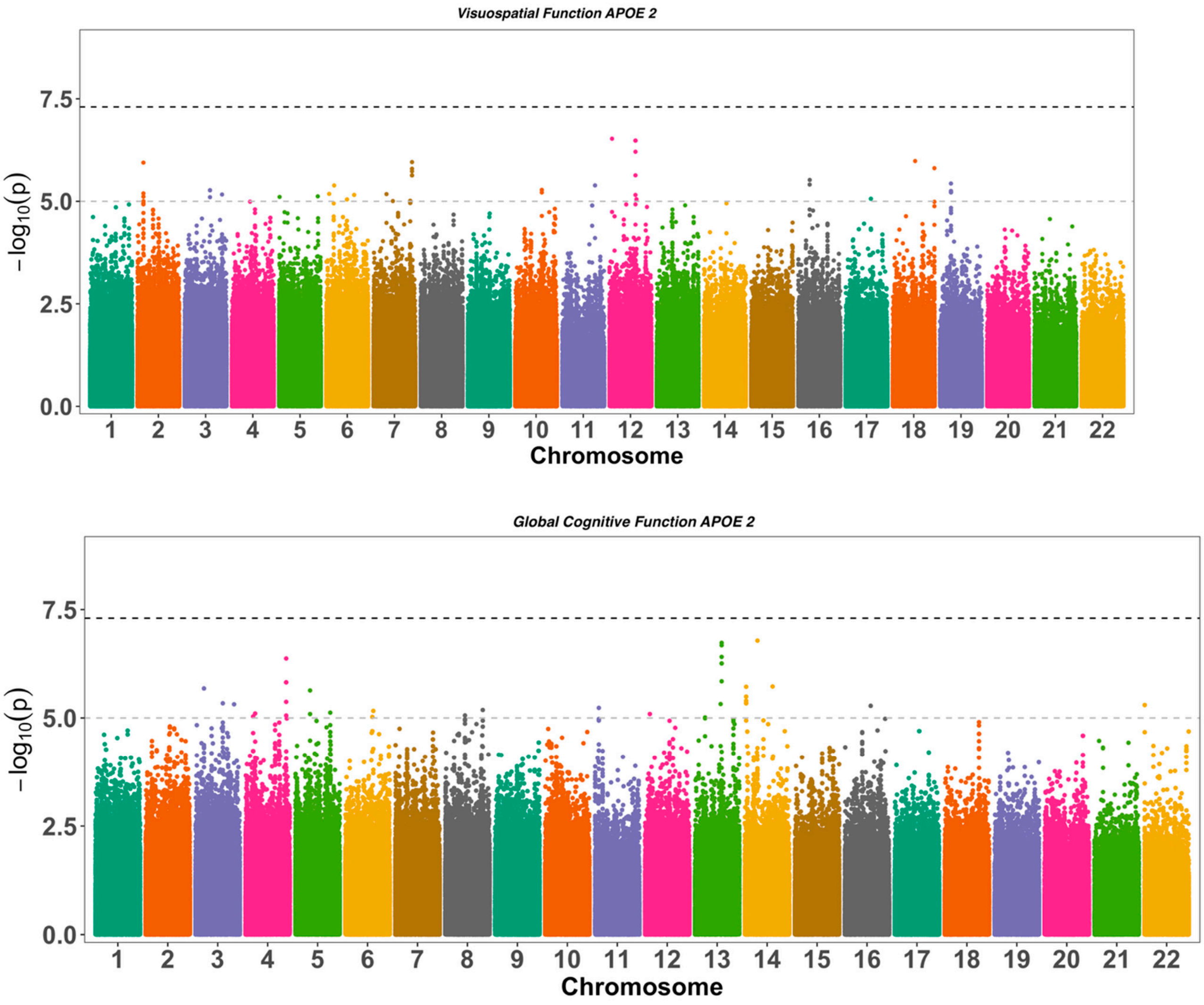

Figure S4: Manhattan plot showing the association of SNPs with the decline of visuospatial function (top) and global function(below) in *APOE2* group. The black dash line indicates the genome-wide significance (p=5.E-08) and grey dash line indicates the suggestive significance of (p=1E-05).

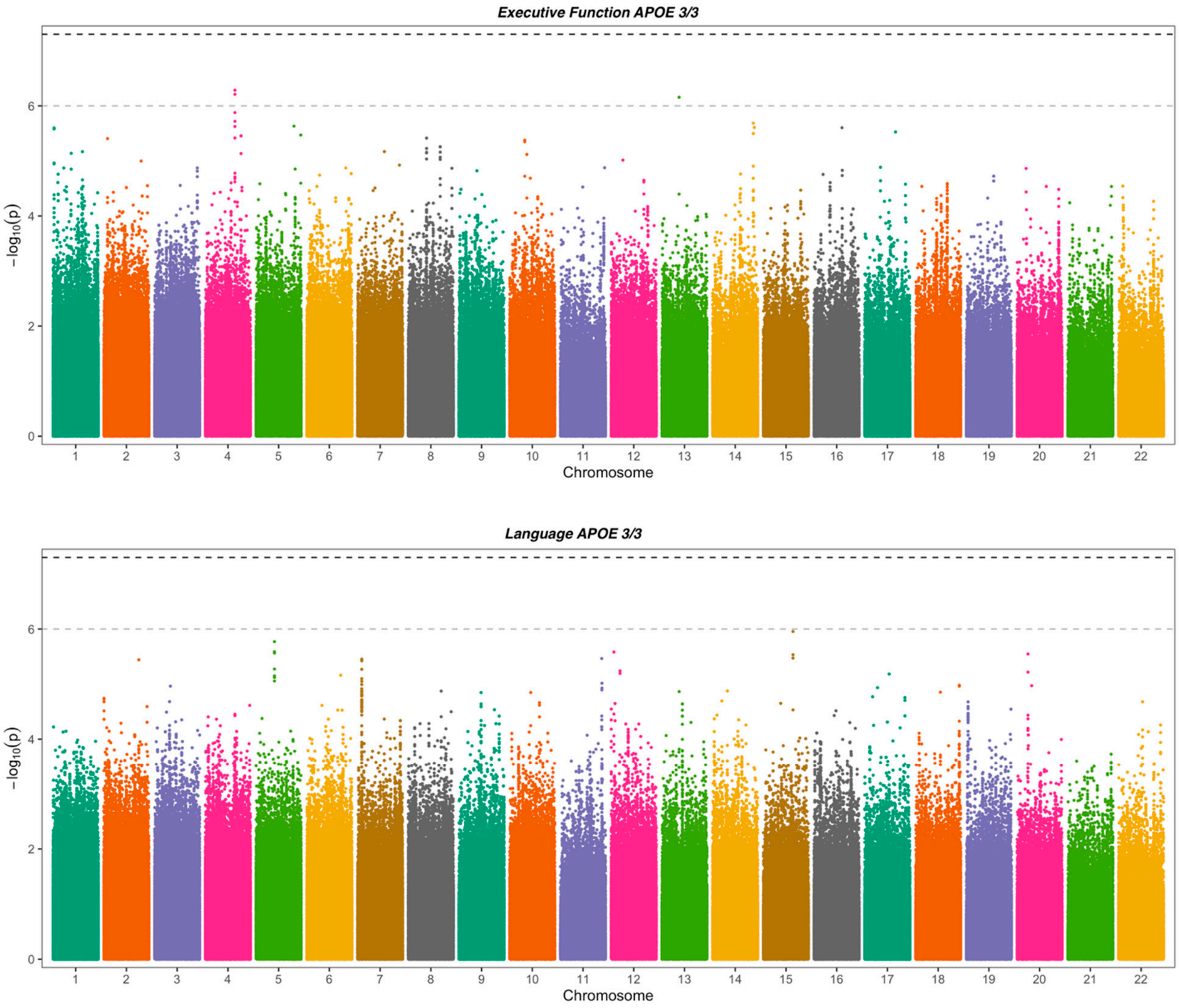

Figure S5: Manhattan plot showing the association of SNPs with the decline of executive function (top) and language (below) in *APOE 3/3* group. The black dash line indicates the genome-wide significance (p=5.E-08) and grey dash line indicates the suggestive significance of (p=1E-05).

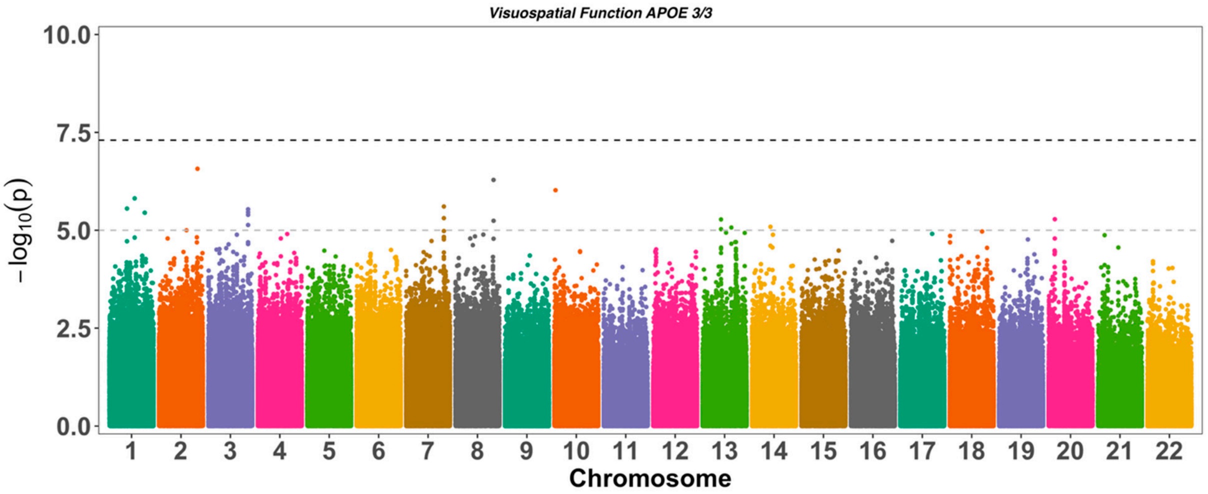

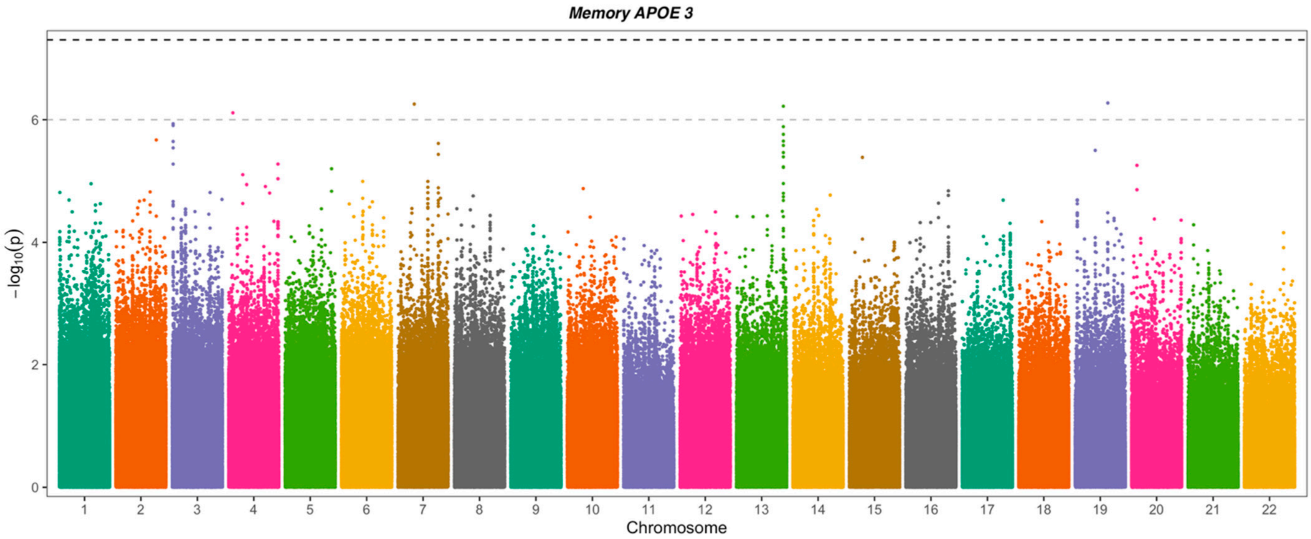

Figure S6: Manhattan plot showing the association of SNPs with the decline of visuospatial function (top) and memory (below) in *APOE 3/3* group. The black dash indicates the genome-wide significance (p=5.E-08) and grey dash line indicates the suggestive significance of (p=1E-05).

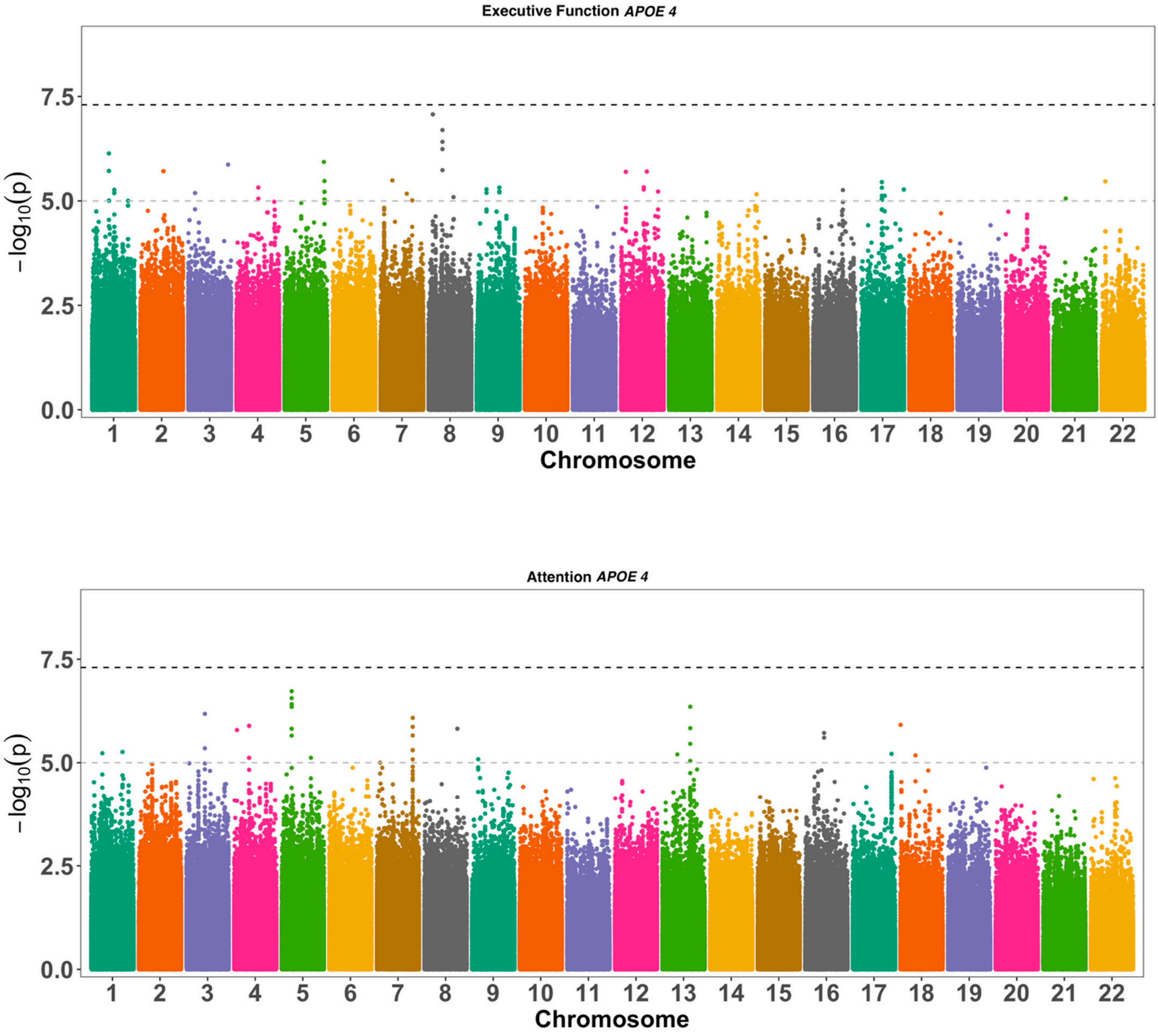

Figure S7: Manhattan plot showing the association of SNPs with decline of executive function (top) and attention (below) in *APOE4* group. The black dash line indicates the genome-wide significance (p=5.E-08) and grey dash line indicates the suggestive significance of (p=1E-05).

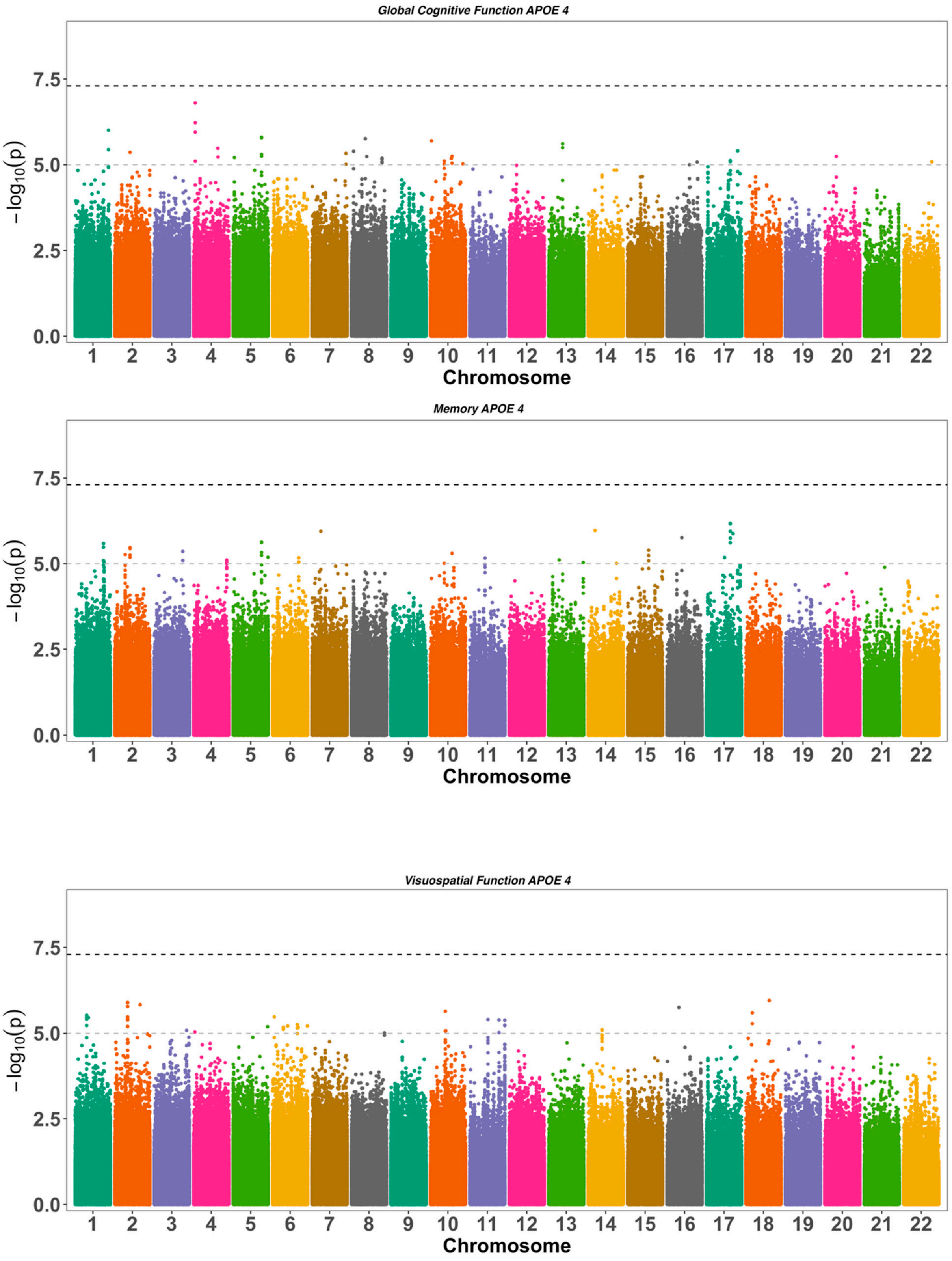

Global Cognitive Function APOE 4
Memory APOE 4
Visuospatial Function APOE 4
$-\log_{10}(p)$
0.0
2.5
5.0
7.5
1 2 3 4 5 6 7 8 9 10 11 12 13 14 15 16 17 18 19 20 21 22
Chromosome

Figure S8: Manhattan plot showing the association of SNPs with the decline of global cognitive function (top) ,memory (middle) and visuospatial function (below) in *APOE*4 group. The black dash line indicates the genome-wide significance (p=5.E-08) and grey dash line indicates the suggestive significance of (p=1E-05).

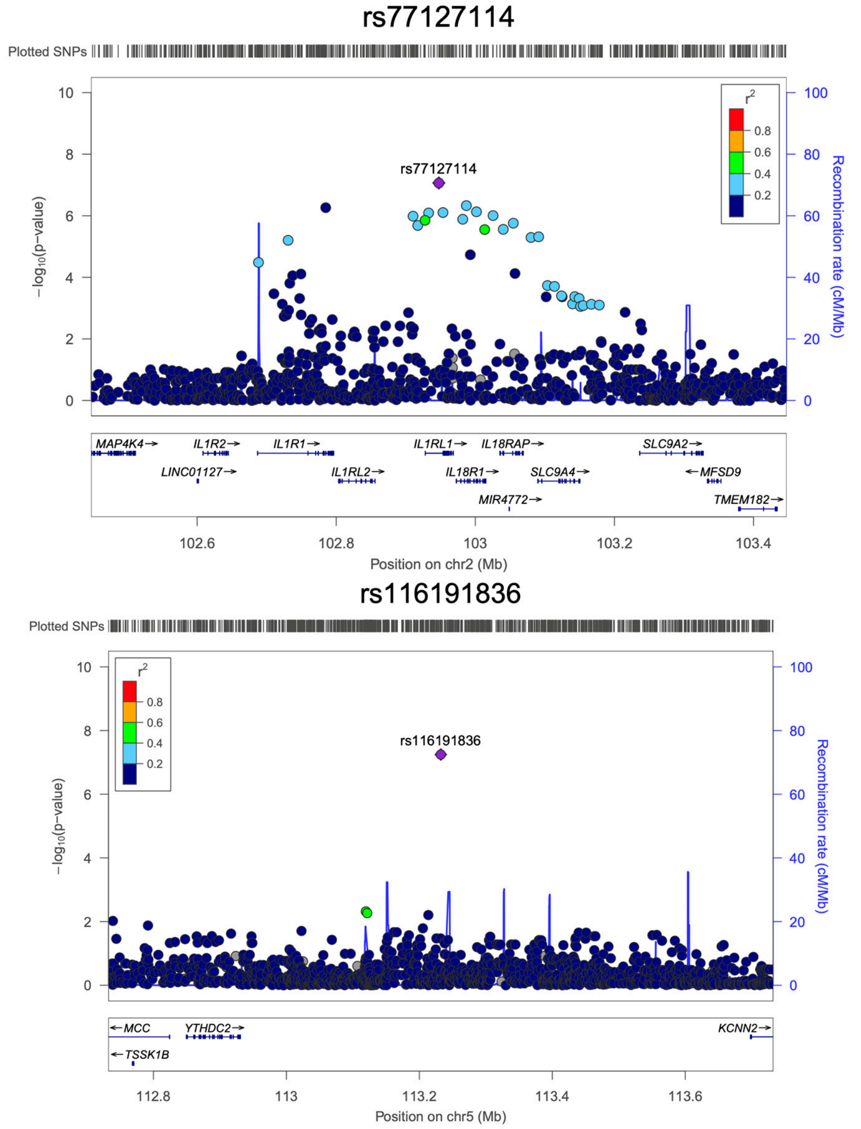

Figure S9: Locuszoom plots showing the association of rs77127114 and rs116191836 with the decline of atttention and language respectively in *APOE2* carrier group.

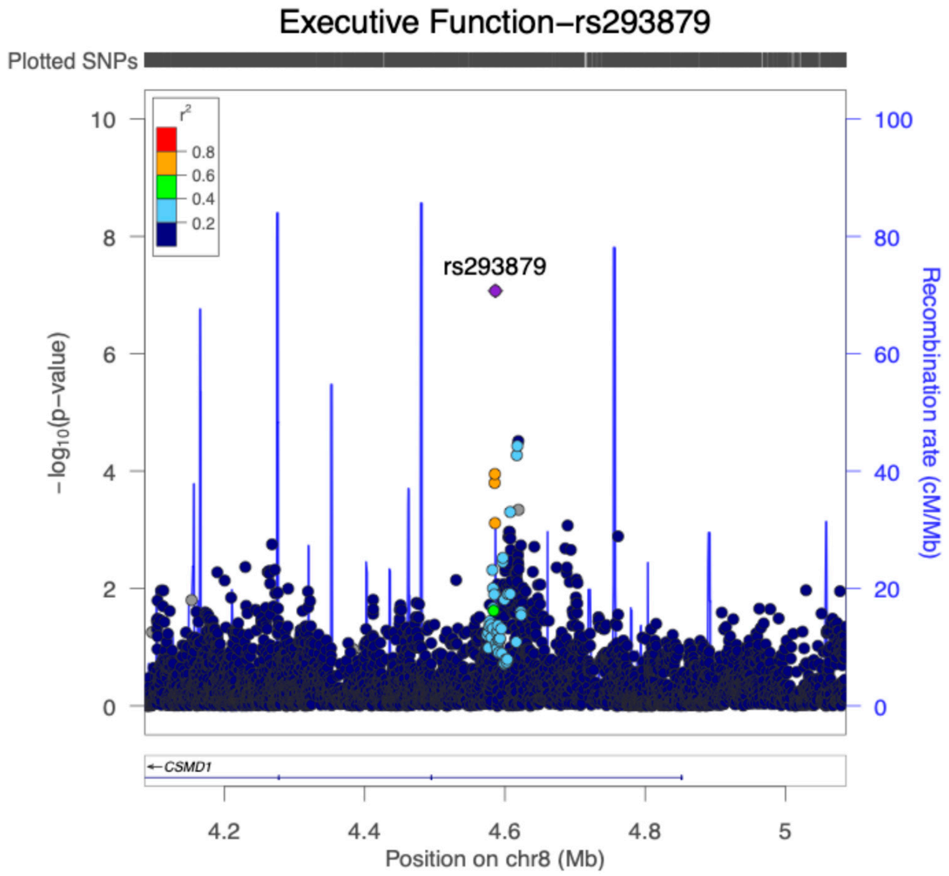

Figure S10: Locuszoom plot showing the association of rs293879 with the decline of executive function in *APOE4* carrier group.
